# Partial Unilateral Lentiginosis: A Systematic Review of 159 Reported Cases and Diagnostic Considerations

**DOI:** 10.7759/cureus.108580

**Published:** 2026-05-10

**Authors:** Marie Line Chedid, Dima Fawaz, Diana Maria Carausu, Jessica Mesquita, Zahraa Awada, Nancy Emmanuel

**Affiliations:** 1 Faculty of Medicine, Medical University of Lodz, Lodz, POL; 2 Faculty of Medicine, Saint Joseph University of Beirut, Beirut, LBN; 3 Faculty of Medicine, Grigore T. Popa University of Medicine and Pharmacy, Iasi, ROU; 4 Department of Dermatology, Clinica Venusta, São Paulo, BRA; 5 Faculty of Medicine, Lebanese University, Beirut, LBN; 6 Department of Dermatology, Faculty of Medicine, University of São Paulo, São Paulo, BRA

**Keywords:** laser therapy, lentiginosis, neurofibromatosis type 1, partial unilateral lentiginosis, pigmentary mosaicism, segmental pigmentation

## Abstract

Partial unilateral lentiginosis is a rare pigmentary disorder characterized by multiple lentigines confined to one side of the body and is frequently misdiagnosed as nevus spilus or segmental neurofibromatosis type 1. Despite its benign nature, its distinctive distribution, often involving visible areas such as the face and neck, may result in cosmetic concern and diagnostic uncertainty.

A systematic review was conducted to synthesize all published cases of partial unilateral lentiginosis. PubMed and Embase were searched from 1983 to 2026 for case reports and case series describing clinically or histopathologically compatible cases. Data on patient demographics, clinical features, associated findings, histopathology, and treatment outcomes were extracted. A total of 159 patients were identified. The condition most commonly presents in childhood, with unilateral lentigines arranged in dermatomal or Blaschko linear patterns. Histopathologic findings were most frequently consistent with lentigo simplex. Reported associations included café-au-lait macules, Lisch nodules, ocular pigmentation, seizures, and rare systemic conditions. The main diagnostic challenge was distinguishing partial unilateral lentiginosis from nevus spilus and mosaic or segmental neurofibromatosis type 1.

Management approaches were variable, with laser-based therapies demonstrating partial to near-complete clearance in small series. Recurrence and post-inflammatory hyperpigmentation, particularly in darker skin phototypes, were notable limitations. This review provides a comprehensive overview of the clinical presentation, associated findings, diagnostic considerations, and treatment outcomes of partial unilateral lentiginosis.

## Introduction and background

Partial unilateral lentiginosis (PUL) is a rare pigmentary disorder characterized by multiple acquired lentigines confined to one side of the body [1-5]. Lesions appear as small, sharply demarcated brown-to-black macules that may be clustered along dermatomal or Blaschko linear patterns, most commonly involving the face, neck, trunk, or limbs [1,4,6-8]. Although benign, the distinctive unilateral distribution frequently leads to diagnostic uncertainty and cosmetic concern.

The terminology surrounding PUL has been inconsistent, with overlapping designations including unilateral lentiginosis, lentiginous mosaicism, segmental lentiginosis, and agminated lentiginosis [9,10]. Clinically, PUL can be difficult to distinguish from nevus spilus and mosaic or segmental neurofibromatosis type 1 (NF1). Unlike nevus spilus, PUL typically lacks a tan or café-au-lait-like background pigmentation, and the interlesional skin is usually normal or only subtly pigmented [1,5,9,11-13], a feature also discussed in prior literature [11]. Although lesions are classically unilateral, limited midline crossing has occasionally been reported, likely reflecting an underlying postzygotic mosaicism [1,3,4,6,14-16].

The etiology of PUL remains incompletely understood. Most evidence supports a postzygotic mosaic disorder involving abnormalities in melanoblast migration or function [1,6,9,15,17-19]. Reports describing associations with café-au-lait macules, Lisch nodules, and other systemic findings have raised consideration of a relationship with mosaic or segmental NF1 [6,7,9,16,18-22]. However, the majority of patients do not fulfill established diagnostic criteria for NF1, supporting the view that PUL may represent a distinct clinical entity rather than a variant within the neurofibromatosis spectrum [1,5,6,13,21,23]. Management of PUL is primarily cosmetic, as no standardized treatment guidelines currently exist. Various laser modalities have demonstrated variable efficacy; however, recurrence and post-inflammatory hyperpigmentation remain important limitations [1,17-19,23,24].

Despite more than four decades of sporadic case reports and small case series, the literature on PUL remains fragmented. No comprehensive synthesis has systematically addressed its clinical spectrum, associated findings, diagnostic challenges, and treatment outcomes. Therefore, the aim of this systematic review is to consolidate all published cases of PUL, summarize its epidemiologic and clinicopathologic features, clarify key diagnostic considerations, and critically evaluate reported treatment outcomes, with particular emphasis on emerging laser-based therapies.

## Review

Methods

Study Design

This systematic review was performed in accordance with the Preferred Reporting Items for Systematic Reviews and Meta-Analyses (PRISMA) 2020 guidelines of published case reports and case series describing PUL. The review focused on synthesizing all published cases of PUL and summarizing its epidemiology, clinical histopathology, and characteristics. The completed PRISMA checklist is provided in Appendix A.

Literature Search Strategy

A comprehensive literature search was performed using the PubMed and Embase databases to identify studies published between 1983 and 2026 reporting cases of PUL. The search strategy used combinations of the following Boolean keywords: ("Partial Unilateral Lentiginosis" OR "Unilateral Lentiginosis" OR "segmental Lentiginosis" OR "Lentiginous mosaicism" OR "agminated lentiginosis" OR "zosteriform lentiginous nevus"). A broad search strategy was intentionally applied to maximize sensitivity due to the rarity of the condition and the limited number of published reports. Filters were set to "English language" and "case series/case reports". Furthermore, the reference lists of all included articles and relevant review papers were manually screened to identify additional eligible publications not captured through the initial database searching.

Eligibility Criteria

Inclusion criteria: Eligible studies consisted of case reports and case series describing patients of any age or sex with clinically and/or histopathologically compatible PUL with a strict clinical definition: "multiple small, well-demarcated brown macules in a segmental/Blaschkoid distribution on a background of normal appearing skin". Studies reporting PUL in isolation or in association with other conditions, including sickle cell anemia, neurofibromatosis, nevi, and ocular findings, were included. Reports describing treated cases, particularly those involving laser-based therapies, were also included to evaluate available treatment outcomes.

Exclusion criteria: Studies were excluded if they described pigmentary disorders without sufficient clinical detail to support a diagnosis of PUL, if they represented review articles, conference abstracts without full text, or non-peer-reviewed sources, or if the language was other than English. Studies were excluded when the overall clinicopathologic diagnosis favored nevus spilus or another melanocytic nevus rather than PUL.

Study Selection

All retrieved records were imported into Rayyan systematic review software (Rayyan Systems Inc., Cambridge, MA, US) for the purpose of screening and duplicate removal. Titles and abstracts were screened independently for relevance and in duplicate by two reviewers, and disagreements were resolved by a third reviewer. 

Data Extraction

Data were extracted from included studies using a standardized data collection form, with the extracted variables as follows: demographics (age and sex), age of onset, clinical presentation, lesion distribution, associated cutaneous and systemic findings, histopathological features, and treatment modalities and reported outcomes, recurrence, and adverse effects when reported. Due to the descriptive nature of the available literature and the predominance of case reports and small case series, a formal quantitative meta-analysis was not performed.

Quality Assessment and Risk of Bias

The methodological quality of the included studies was assessed using the Joanna Briggs Institute (JBI) critical appraisal checklist for case reports and case series. Two reviewers independently assessed each study for risk of bias, and discrepancies were resolved by a third reviewer. Studies were evaluated based on the clarity of demographic data, clinical history, diagnostic tests, and follow-up.

Data Synthesis

Due to the descriptive nature and heterogeneity of the available data, results were synthesized qualitatively, and descriptive statistics were used where appropriate to summarize patient demographics, clinical features, and treatment outcomes. Findings were then organized into domains such as epidemiology, clinical presentation, histopathology, differential diagnosis, and treatment approaches. Missing data (e.g., specific histopathological stains or long-term follow-up) were handled via available case analysis, where percentages were calculated based only on the subset of studies reporting that specific variable. Treatment outcomes were summarized and tabulated to allow comparison across reported modalities.

Results 

Study Selection

**Figure 1:**
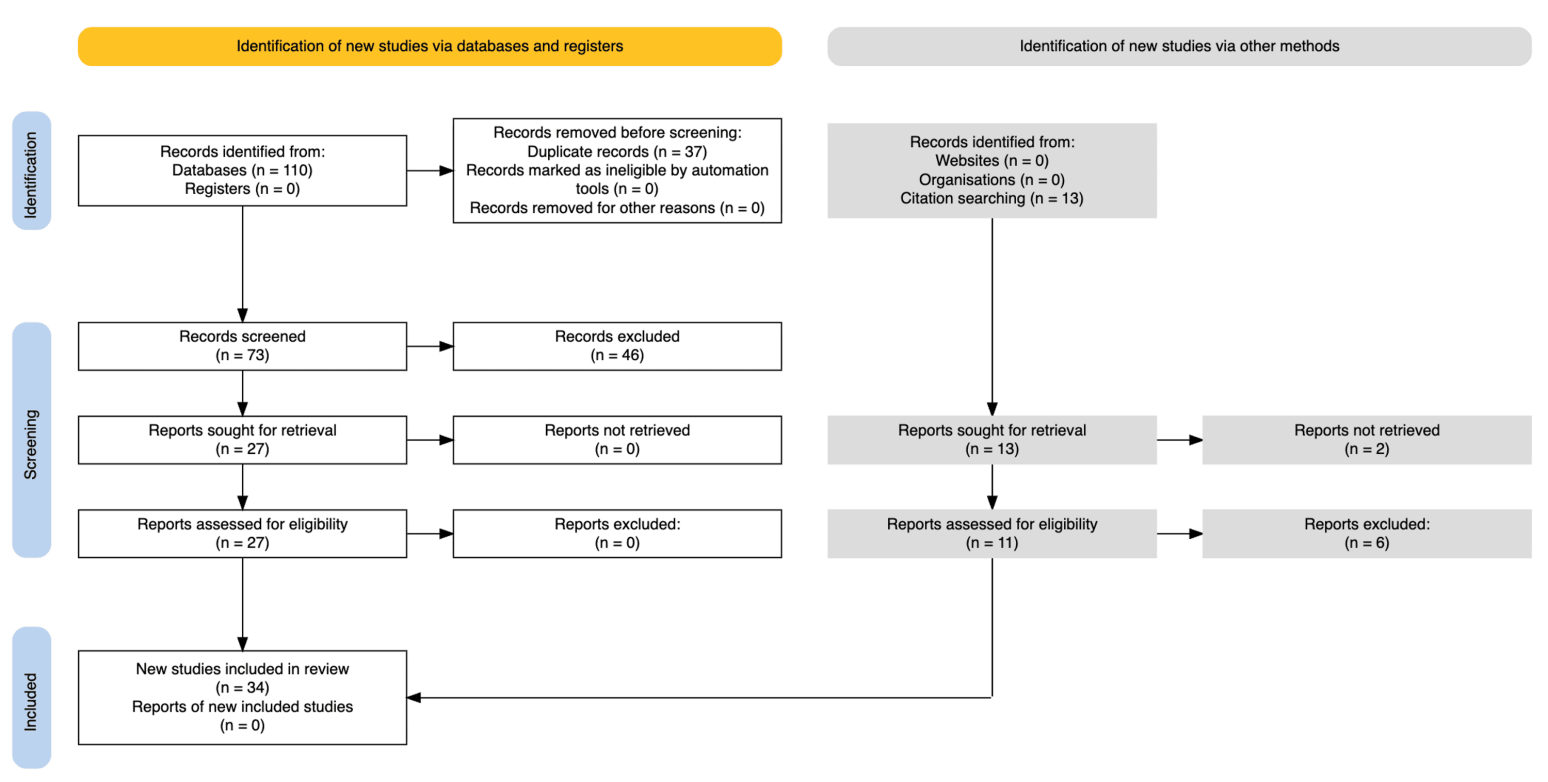
PRISMA 2020 flow diagram for the systematic review of PUL PRISMA, Preferred Reporting Items for Systematic Reviews and Meta-Analyses; PUL, partial unilateral lentiginosis.

The initial literature search yielded a total of 110 records from PubMed and Embase. Following the removal of 37 duplicates, 73 titles and abstracts were screened for relevance, of which 27 met the predefined inclusion criteria. An additional supplemental search using expanded keywords ('agminated lentiginosis', 'zosteriform lentiginous nevus', and 'segmental lentiginosis') identified 13 additional records. After full-text review and application of strict exclusion criteria (e.g., absence of full text or histopathological evidence of nevus cell nests), five of these records were included. These additions, along with two additional articles regarding differential diagnosis and laser treatment, resulted in a total of 34 articles eligible for final qualitative synthesis. The study selection process is summarized in the PRISMA 2020 flow diagram (Figure 1).

Study Characteristics

The included studies consisted mainly of case reports and case series, representing a total of 159 PUL patients. The data originated from several geographic regions across Asia, Europe, and North America, reflecting the sporadic international reporting of this rare disorder. Overall, most included studies adequately reported demographic, clinical, and diagnostic information; however, follow-up adverse events and standardized treatment outcomes were inconsistently reported. A summary of the risk-of-bias assessment for the included studies is provided in Appendices B, C.

Demographic and Epidemiologic Findings

Obtaining epidemiological insights into rare diseases such as PUL is challenging due to the limited literature and the high likelihood of unbiopsied or misdiagnosed cases [5,6,13,14]. Both females and males may be affected. Among the 159 reported PUL cases, age at presentation ranged from 5 months to 86 years old, with an approximate mean age of 14.2, noting that one study reported median age, which may limit precision. A female predominance was observed, with 114 females and 45 males (female-to-male ratio 2.53:1), consistent with previously reported series [3,6]. Data regarding racial or ethnic distribution remain limited. While Palleschi et al. reported a predominance of Caucasian patients [4], Kim et al. described cases primarily originating from Korea, Japan, Turkey, India, and Spain [1]. In our review, most reported cases originated from Asia and Europe, likely reflecting regional reporting patterns rather than true epidemiologic differences. The main demographic, clinical, histopathological, and associated features of reported cases are summarized in Table 1, while detailed case-level characteristics are provided in Appendix D.

**Table 1:** Summary of clinical characteristics of reported partial unilateral lentiginosis cases (n=159) CNS, central nervous system; NF1, neurofibromatosis type 1.

Characteristics	Findings
Number of patients	159
Sex distribution	Female: 114 (71.7%)/male: 45 (28.3%)
Age at presentation	Ranged from 5 months to 86 years; mean age approximately 14.2
Age of onset	Congenital or early childhood within the first decade of life, although an occasional onset in adolescence or early adulthood has been reported.
Laterality	Predominantly unilateral with sharp midline demarcation; rare bilateral segmental involvement reported
Distribution pattern	Segmental, dermatomal, and/or Blaschko linear following mosaic pattern
Most commonly affected sites	Face, neck, trunk, and upper extremities
Lower extremity involvement	Less frequent
Lesion morphology	Multiple, small, well demarcated brown macules, typically clustered in a segmental pattern
Background pigmentation	Typically absent or minimal; however, rare cases occur with nevoid hypopigmentation (mosaicism/twin-spotting)
Histopathology	Lentigo simplex pattern (increased basal melanocytes without nevus cell nests)
Associated cutaneous findings	Café-au-lait macules, freckles, and melanocytic nevi (in minority of cases)
Ocular findings	Ipsilateral ocular pigmentation or Lisch nodules (rare)
Systemic associations	Seizures, CNS abnormalities, sickle cell anemia (isolated case reports), and rare malignant transformation (melanoma)
Association with NF1	Most patients did not meet NIH diagnosis criteria for NF1, but a subset of cases may represent a mosaic or segmental form of NF1, especially when accompanied by ipsilateral NF1 stigmata.

Clinical Features

PUL manifests as multiple small, well-demarcated brown macules confined to one side of the body, often following Blaschko lines or dermatomal patterns, most commonly affecting the face, neck, trunk, and limbs [1,4,6,7,25]. The upper extremities are affected more frequently than the lower extremities, and extensive forms may involve an entire side of the body [6,9,14,18,23]. Clinically, lesions consist of numerous small, flat, non-hairy brown macules measuring 1-5 mm in diameter with well-defined borders and variable pigmentation [25-29]. However, larger pigmented lesions exceeding 15 mm, resembling CALMs, may occasionally coexist within the lentiginous area [30-32]. Lesions are typically asymptomatic, appear at birth or during early childhood, and may darken or expand gradually over time [2,12,14,15,19,24,27]. Rare reports have described melanoma occurring within areas of segmental lentiginosis; however, the causal relationship remains uncertain, and these findings may represent coincidental or collision lesions rather than true malignant transformation of PUL [29,30].

Histopathology

Histopathological examination most frequently demonstrates features of lentigo simplex, characterized by a marked increase in typical melanocytes (hyperplasia) and melanin content within the basal layer [1,5,7,31]. Characteristic findings include regular elongation of epidermal rete ridges and the strict absence of nevus cell nests (theques) at the dermoepidermal junction [1,2,4,7,27,28,31]. While the pure lentiginous pattern is standard, a jentigo pattern featuring small nests of pigment-containing nevus cells at dermal papillae tips has been reported as a mixed histological variant or the second stage of lesion evolution [3,4,7,29]. The skin between macules often exhibits mild-to-moderate hyperpigmentation and increased basal melanin compared to unaffected skin, suggesting a localized field effect [1,6,8]. Positive immunohistochemical staining for PKC-βII confirms active melanogenesis within lesions. Ultrastructural analysis (electron microscopy) reveals single melanocytes at the dermoepidermal junction containing melanosomes in various developmental stages [1,26]. Histological confirmation is essential to distinguish PUL from nevus of Ota (which shows dermal melanocytosis), nevus spilus (which typically features junctional or compound nevi nests), and solar lentigo (which is characterized by dermal solar elastosis) [7,25,28].

Associated Findings

While many patients with PUL exhibit no extracutaneous abnormalities, a diverse range of cutaneous and systemic associations has been documented across the 159 cases. The most frequent association is with café-au-lait macules (CALMs), often appearing within the same segmental distribution as the lentigines. Other documented features include axillary or inguinal freckling, Lisch nodules, and rare cutaneous neurofibromas [1,3,6,9,16]. Beyond Lisch nodules, findings include bulbar conjunctival or uveal pigmentation, ocular nevi (iris or caruncula), and isolated reports of congenital cataracts [1,6,7,9].

Reported central nervous system (CNS) associations include seizures (focal or generalized), mental retardation, intracranial vascular malformations, and rare low-grade gliomas such as pilocytic astrocytoma [1,5,7,9]. Rare systemic associations include sickle cell anemia, celiac disease, and musculoskeletal abnormalities. Crucially, while PUL is primarily benign, a single case documented the development of superficially spreading melanoma and melanoma in situ within the PUL distribution in an 86-year-old patient [1,7,9,19,30].

Treatment Outcomes

While PUL is medically benign, management is frequently sought for aesthetic concerns. Clinical outcomes reported across the 34 included studies indicate that topical agents (hydroquinone or tretinoin) and chemical peels are consistently unsuccessful. Cryotherapy has shown efficacy in isolated cases but carries risks of scarring and permanent hypopigmentation [9,23-25]. Q-switched (QS) lasers are the most documented intervention. Low-fluence QS Nd:YAG lasers achieved >50% improvement in 62.5% to 100% of patients in small Korean cohorts. QS Alexandrite lasers also provided substantial clearance in various phototypes [1,18,23].

Dual-wavelength copper bromide (511 nm/578 nm) and 532 nm fractional picosecond lasers have demonstrated high patient satisfaction and significant pigment reduction in recent case reports [17,24]. Complications are generally transient, including erythema and edema. More significant risks include mottled hypopigmentation or post-inflammatory hyperpigmentation (PIH), particularly in patients with darker Fitzpatrick skin types [1,18,19,23].

Discussion

PUL represents a rare pigmentary disorder that remains underrecognized and frequently misdiagnosed due to its clinical overlap with other segmental pigmentary disorders such as nevus spilus and mosaic or NF1. This review consolidates the understanding of the characteristics, presentations, associated findings, and treatment outcomes reported in PUL patients. 

Physiopathology

PUL is generally considered a form of postzygotic pigmentary mosaicism resulting from mutations affecting neural crest-derived melanoblasts. While the exact genetic triggers remain under investigation, the localized nature of the lesions supports a non-neoplastic proliferation of melanocytes [1,6,9,17,18].

A significant advancement in understanding PUL's origin is the theory of non-allelic twin spotting (didactic mosaicism). This phenomenon explains rare cases where PUL coexists with nevoid hypopigmentation (such as nevus depigmentosus). In this model, a single somatic recombination event in a progenitor cell produces two distinct mutant daughter cell lines: one with a propensity for increased melanin production and another with hypofunctional melanocytes [1,4,15,33,34].

PUL and NF1

The relationship between PUL and NF1 is a major point of clinical debate. While some authors propose that PUL represents a segmental form of NF1, most patients do not fulfill the NIH diagnostic criteria for the systemic disease. The presence of ipsilateral Lisch nodules or neurofibromas in a minority of cases suggests a possible relationship with mosaic NF1, but no definitive genetic link has been established across the broader PUL population. Therefore, while careful screening for NF1 stigmata is mandatory, PUL is currently classified as a distinct clinical entity [1,6,16,19,22,25,32].

PUL and CNS Abnormalities

The association of PUL with seizures, cognitive impairment, and ipsilateral cerebrovascular malformations suggests a potential shared developmental origin involving neural crest derivatives. Although these findings are limited to isolated case reports, they highlight the necessity of a neurological history in patients with extensive or facial lentiginosis [9,13,16,19].

Associated Cutaneous and Systemic Findings

PUL frequently coexists with other integumentary manifestations, including vitiligo, blue nevi, and melanocytic nevi. The unusual association with alopecia universalis suggests a broader disruption of neuroectodermal derivatives affecting both hair follicles and melanocytes. Similarly, ocular and periocular pigmentary abnormalities, such as uveal pigmentation or ocular nevi, indicate that the mosaicism in PUL may extend beyond the epidermis [1,3,9,25,27,32].

Although PUL is considered a benign condition, isolated reports have described melanoma occurring within areas of segmental lentiginosis. However, a direct causal relationship has not been established, and these cases likely represent coincidental or collision phenomena. Nevertheless, careful clinical monitoring of atypical or evolving lesions remains prudent. This rare but significant risk of malignant transformation necessitates long-term clinical surveillance, particularly in older patients or those with atypical lesion evolution. Additionally, rare systemic associations such as sickle cell anemia have been reported; while likely coincidental, they reinforce the importance of a comprehensive systemic evaluation [5,7,10,26,30].

Diagnosis and Differential Considerations

Diagnosis is primarily clinical, supplemented by dermoscopy and selective histopathology. The most essential distinction is from nevus spilus, which is characterized by a tanned background patch and the histological presence of nevus cell nests, features strictly absent in PUL [1,7,8,24].

Clinicians must also distinguish PUL from inherited multisystem syndromes such as LEOPARD or Peutz-Jeghers, which typically present with bilateral involvement and distinct systemic anomalies. Given the diagnostic challenges, applying the NIH criteria for NF1 (Table 2) remains the standard for excluding systemic neurofibromatosis [6,9,12,13,23,32].

**Table 2:** NIH diagnostic criteria for neurofibromatosis type 1 (NF1) used to exclude NF1 in patients with partial unilateral lentiginosis

NIH criteria for the diagnosis of neurofibromatosis type 1	
1	Six or more cafe-au-lait skin macules >5 mm in prepubertal individuals and >15 mm in postpubertal individuals
2	Two or more neurofibromas of any type or one plexiform neurofibroma
3	Axillary or inguinal freckling
4	Two or more Lisch nodules
5	Optic glioma
6	Bone lesion with sphenoid dysplasia or thinning of the long bone cortex with or without pseudarthrosis
7	A first-degree relative (parent, sibling, or offspring) that meets NIH criteria

**Figure 2:**
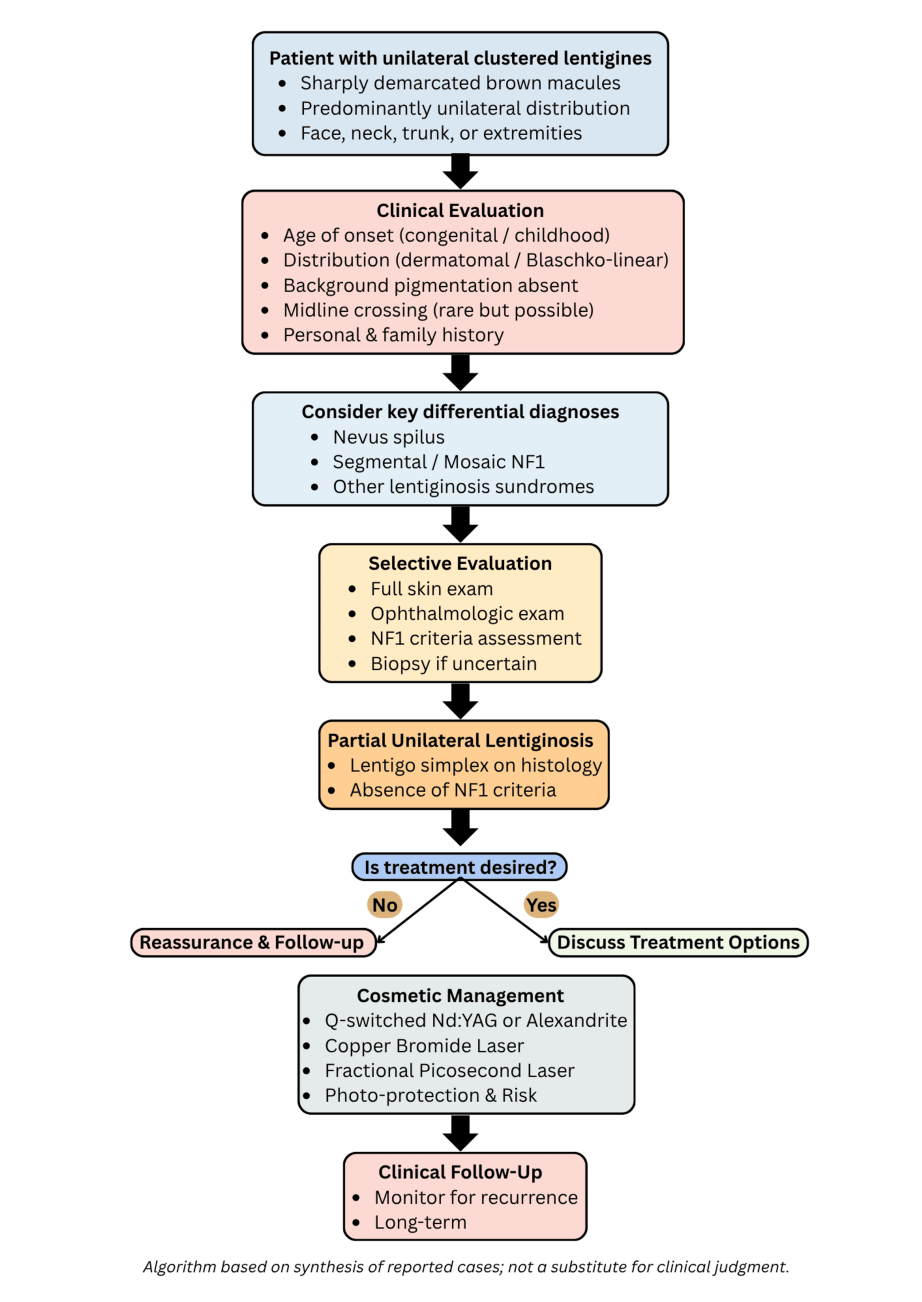
Proposed diagnostic and management algorithm for partial unilateral lentiginosis The algorithm emphasizes clinical evaluation and exclusion of key differential diagnoses. Additional investigations are intended to be performed selectively based on clinical findings and are not recommended routinely in asymptomatic patients. NF1, neurofibromatosis type 1.

Based on a synthesis of reported cases, we propose a pragmatic diagnostic and management algorithm to assist clinicians in distinguishing PUL from its principal differential diagnoses and guiding cosmetic treatment decisions. Based on the synthesis of reported cases, we propose a pragmatic diagnostic algorithm to assist clinicians in distinguishing PUL from its principal differential diagnoses, summarized in Figure 2.

Treatment Considerations and Challenges

Management of PUL is elective and primarily addresses cosmetic concerns, as there is currently no standardized medical treatment. This review highlights a clear shift from traditional topicals and cryotherapy, which are largely ineffective or associated with risks of scarring and permanent hypopigmentation, toward advanced pigment-targeting laser therapies. However, several clinical challenges complicate the management of these patients: PUL often appears more resistant to laser therapy compared with other agminated pigmentary lesions. High recurrence rates remain a major limitation; studies have documented partial repigmentation in nearly all patients within months of the final session, frequently necessitating repeated "booster" treatments to maintain results [8,18].

The risk of mottled hypopigmentation or PIH is significant, particularly in patients with darker skin phototypes (Fitzpatrick IV-VI). For these individuals, a conservative approach using lower fluences or newer fractional picosecond modalities is essential to minimize adverse effects [1,6,18,19,23,24].

Overall, these observations support an individualized, phototype-guided approach to cosmetic management in PUL. Reported treatment modalities and outcomes are summarized in Table 3, with detailed parameters provided in Appendix E.

**Table 3:** Summary of reported treatment outcomes in partial unilateral lentiginosis PUL, partial unilateral lentiginosis.

Treatment modality	Number of patients	Reported outcomes	Adverse effects/limitations	Ref.
Low fluence, Q-switched Nd:YAG laser (1,064 nm)	11	High initial improvement. Partial to near-complete clearance in most treated cases	High rate of recurrence; side effects include: mottled hypopigmentation, mild erythema, and transient burning	[6,21]
Q-switched Alexandrite laser (755 nm)	2	Patient 1 (phototype III) achieved substantial clearance. Patient 2 (phototype V) saw 50% partial improvement.	Phototype V patient developed PIH, which was managed by hydroquinone.	[23]
Mixed laser modalities (GS Nd:YAG, IPL, Alexandrite, and others)	43	Variable results, some achieved >50% improvement; however, a larger retrospective analysis reported a very poor response with none achieving >50% improvement.	Transient post-inflammatory hyperpigmentation (PIH) is frequently observed (after 532 nm). PUL appears more resistant to these lasers.	[1,8]
Copper bromide laser (511 nm and 578 nm)	1	Significant fading and brighter facial skin were achieved after two sessions, with sustained six months follow-up.	No side effects were observed. Limited evidence; single case	[17]
Fractional picosecond laser Nd:YAG (532 nm)	1	Marked improvement after four sessions with high patient satisfaction	Well tolerated; side effects were limited to mild, transient erythema and a burning sensation that resolved within an hour.	[24]
Cryotherapy (liquid nitrogen)	2	Patient 1: successful removal of lesions after four sessions. Patient 2: cryosurgery was unsuccessful and resulted in recurrence or failure.	Potential for transient hypopigmentation, scarring, or deep penetration	[9,25]
Topical agents and chemical peels	2	Generally unsuccessful. Glycolic acid peels failed to improve lesions in one patient before switching to cryotherapy. Another patient saw no improvement or recurrence using a combination of hydroquinone, glycolic acid, and corticosteroids.	Ineffective for long term clearance of PUL	[9,25]

Clinicians should prioritize managing patient expectations regarding the likelihood of partial response and the high probability of recurrence. In many asymptomatic cases, clinical observation and strict photoprotection remain the most prudent management strategies [8,17,19].

Limitations

The primary limitation of this systematic review is the scarcity of published cases of PUL, which restricts the ability to draw robust epidemiological conclusions and limits comprehensive analyses of age of onset, lesion characteristics, and clinical spectrum. In addition, many cases were likely unbiopsied or misdiagnosed, further contributing to underrepresentation in the literature. The unresolved and frequently debated relationship between PUL and its differential diagnoses, particularly NF1, also complicates interpretation and prevents the establishment of universally accepted diagnostic criteria. The absence of molecular or genetic confirmation in most reported cases limits deeper insight into the underlying pathogenesis. Regarding treatment, the small number of treated patients, heterogeneity in laser parameters, and inconsistent reporting of outcomes and follow-up durations limit meaningful comparisons between therapeutic modalities and preclude assessment of long-term efficacy and recurrence rates. Nevertheless, systematic data extraction and tabulation of available evidence allowed for a structured synthesis while minimizing interpretive bias.

## Conclusions

PUL is a rare pigmentary mosaicism characterized by unilateral clusters of lentigines following segmental or Blaschko linear patterns. While its pathophysiology involving postzygotic abnormalities remains incompletely understood, diagnosis relies on distinguishing PUL from nevus spilus and NF1 through clinical evaluation and histopathology. Although associated with isolated ocular or neurologic findings, most patients do not meet NF1 criteria, supporting PUL as a distinct clinical entity. Management is primarily cosmetic, with QS, copper bromide, and fractional picosecond lasers showing encouraging results. However, high recurrence and the risk of PIH, particularly in darker skin phototypes, necessitate cautious treatment selection and photoprotection.

Overall, this systematic review consolidates fragmented evidence on PUL, clarifies key diagnostic considerations, and summarizes available treatment outcomes. Recognizing PUL may reduce patient anxiety and support informed aesthetic planning, though further molecular studies are needed to establish standardized diagnostic and therapeutic guidelines.

## Appendices

Appendix A 

**Table 4:** PRISMA 2020 checklist

Section and topic	Item #	Checklist item	Location where the item is reported
TITLE			
Title	1	Identify the report as a systematic review.	Title
ABSTRACT			
Abstract	2	See the PRISMA 2020 for Abstracts checklist.	Abstract
INTRODUCTION			
Rationale	3	Describe the rationale for the review in the context of existing knowledge.	Introduction
Objectives	4	Provide an explicit statement of the objective(s) or question(s) the review addresses.	Introduction
METHODS			
Eligibility criteria	5	Specify the inclusion and exclusion criteria for the review and how studies were grouped for the syntheses.	Methods
Information sources	6	Specify all databases, registers, websites, organizations, reference lists, and other sources searched or consulted to identify studies. Specify the date when each source was last searched or consulted.	Methods
Search strategy	7	Present the full search strategies for all databases, registers, and websites, including any filters and limits used.	Methods
Selection process	8	Specify the methods used to decide whether a study met the inclusion criteria of the review, including how many reviewers screened each record and each report retrieved, whether they worked independently, and, if applicable, details of automation tools used in the process.	Methods
Data collection process	9	Specify the methods used to collect data from reports, including how many reviewers collected data from each report, whether they worked independently, any processes for obtaining or confirming data from study investigators, and, if applicable, details of automation tools used in the process.	Methods
Data items	10a	List and define all outcomes for which data were sought. Specify whether all results that were compatible with each outcome domain in each study were sought (e.g., for all measures, time points, analyses), and if not, the methods used to decide which results to collect.	Methods
	10b	List and define all other variables for which data were sought (e.g., participant and intervention characteristics, funding sources). Describe any assumptions made about any missing or unclear information.	Methods
Study risk of bias assessment	11	Specify the methods used to assess risk of bias in the included studies, including details of the tool(s) used, how many reviewers assessed each study, and whether they worked independently, and if applicable, details of automation tools used in the process.	Methods
Effect measures	12	Specify for each outcome the effect measure(s) (e.g., risk ratio, mean difference) used in the synthesis or presentation of results.	Methods
Synthesis methods	13a	Describe the processes used to decide which studies were eligible for each synthesis (e.g., tabulating the study intervention characteristics and comparing against the planned groups for each synthesis (item #5)).	Methods
	13b	Describe any methods required to prepare the data for presentation or synthesis, such as handling of missing summary statistics or data conversions.	Methods
	13c	Describe any methods used to tabulate or visually display the results of individual studies and syntheses.	Methods
	13d	Describe any methods used to synthesize results and provide a rationale for the choice(s). If meta-analysis was performed, describe the model(s), method(s) to identify the presence and extent of statistical heterogeneity, and software package(s) used.	Methods
	13e	Describe any methods used to explore possible causes of heterogeneity among study results (e.g., subgroup analysis, meta-regression).	Methods
	13f	Describe any sensitivity analyses conducted to assess the robustness of the synthesized results.	Methods
Reporting bias assessment	14	Describe any methods used to assess the risk of bias due to missing results in a synthesis (arising from reporting biases).	Methods
Certainty assessment	15	Describe any methods used to assess certainty (or confidence) in the body of evidence for an outcome.	Methods
RESULTS			
Study selection	16a	Describe the results of the search and selection process, from the number of records identified in the search to the number of studies included in the review, ideally using a flow diagram.	Results, Figure 1
	16b	Cite studies that might appear to meet the inclusion criteria, but which were excluded, and explain why they were excluded.	Results
Study characteristics	17	Cite each included study and present its characteristics.	Results, Table 1, Supplementary Table S1
Risk of bias in studies	18	Present assessments of risk of bias for each included study.	Methods, Results
Results of individual studies	19	For all outcomes, present, for each study: (a) summary statistics for each group (where appropriate) and (b) an effect estimate and its precision (e.g., confidence/credible interval), ideally using structured tables or plots.	Results, Table 1
Results of syntheses	20a	For each synthesis, briefly summarise the characteristics and risk of bias among contributing studies.	Results
	20b	Present the results of all statistical syntheses conducted. If a meta-analysis was done, present, for each, the summary estimate and its precision (e.g., confidence/credible interval), and measures of statistical heterogeneity. If comparing groups, describe the direction of the effect.	Methods
	20c	Present the results of all investigations of possible causes of heterogeneity among study results.	Results
	20d	Present the results of all sensitivity analyses conducted to assess the robustness of the synthesized results.	Methods
Reporting biases	21	Present assessments of risk of bias due to missing results (arising from reporting biases) for each synthesis assessed.	Methods
Certainty of evidence	22	Present assessments of certainty (or confidence) in the body of evidence for each outcome assessed.	Methods
DISCUSSION			
Discussion	23a	Provide a general interpretation of the results in the context of other evidence.	Discussion
	23b	Discuss any limitations of the evidence included in the review.	Discussion
	23c	Discuss any limitations of the review processes used.	Discussion
	23d	Discuss implications of the results for practice, policy, and future research.	Discussion
OTHER INFORMATION			
Registration and protocol	24a	Provide registration information for the review, including register name and registration number, or state that the review was not registered.	Methods
	24b	Indicate where the review protocol can be accessed, or state that a protocol was not prepared.	Methods
	24c	Describe and explain any amendments to information provided at registration or in the protocol.	Methods
Support	25	Describe sources of financial or non-financial support for the review, and the role of the funders or sponsors in the review.	Disclosure section
Competing interests	26	Declare any competing interests of review authors.	Disclosure section
Availability of data, code, and other materials	27	Report which of the following are publicly available and where they can be found: template data collection forms; data extracted from included studies; data used for all analyses; analytic code; any other materials used in the review.	Appendices

Appendix B 

**Table 5:** Methodological quality assessment of included case reports using the JBI critical appraisal checklist

Study	Q1 demo	Q2 history	Q3 clinical	Q4 diagnostics	Q5 intervention	Q6 post-intervention	Q7 adverse events	Q8 takeaway	Overall
González-Sixto [30]	Y	Y	Y	Y	Y	Y	U	Y	Include
Lee et al. [31]	Y	Y	Y	Y	NA	NA	U	Y	Include
Lee et al. [32]	Y	Y	Y	Y	Y	U	U	Y	Include
Bayramgürler et al. [33]	Y	Y	Y	Y	NA	NA	U	Y	Include
Kabashima et al. [34]	Y	Y	Y	Y	NA	NA	U	Y	Include
Hughes et al [26]	Y	Y	Y	Y	NA	NA	U	Y	Include
Holder et al. [10]	Y	Y	Y	Y	NA	NA	U	Y	Include
Parslew et al. [13]	Y	Y	Y	Y	NA	NA	U	Y	Include
Lee et al. [21]	Y	Y	Y	Y	NA	NA	U	Y	Include
Schaffer et al. [9]	Y	Y	Y	Y	Y	Y	U	Y	Include
Chen et al. [16]	Y	Y	Y	Y	NA	NA	U	Y	Include
Kim et al. [14]	Y	Y	Y	Y	NA	NA	U	Y	Include
Palleschi et al. [4]	Y	Y	Y	Y	NA	NA	U	Y	Include
Serarslan G [25]	Y	Y	Y	Y	Y	Y	U	Y	Include
In and Kang [15]	Y	Y	Y	Y	NA	NA	U	Y	Include
González-sixto [28]	Y	Y	Y	Y	NA	NA	U	Y	Include
Kim et al. [19]	Y	Y	Y	Y	Y	Y	Y	Y	Include
Gupta et al. [7]	Y	Y	Y	Y	Y	U	U	Y	Include
Erdem et al. [20]	Y	Y	Y	Y	Y	U	U	Y	Include
Khalil et al. [12]	Y	Y	Y	U	NA	NA	U	Y	Include
Hindritiani et al. [17]	Y	Y	Y	Y	Y	Y	Y	Y	Include
Lueangarun et al. [24]	Y	Y	Y	Y	Y	Y	Y	Y	Include

Appendix C 

**Table 6:** Methodological quality assessment of included case series using the JBI critical appraisal checklist

Study	Q1 inclusion	Q2 standard measurement	Q3 valid diagnosis	Q4 consecutive	Q5 complete	Q6 demographics	Q7 clinical info	Q8 outcomes/follow-up	Q9 site info	Q10 stats	Overall
Trattner et al. [5]	Y	Y	Y	U	Y	Y	Y	Y	Y	NA	Include
Piqué et al. [3]	Y	Y	Y	U	Y	Y	Y	Y	Y	NA	Include
Romiti et al. [2]	Y	Y	Y	U	Y	Y	Y	Y	Y	NA	Include
Pretel et al. [23]	Y	Y	Y	U	Y	Y	Y	Y	Y	NA	Include
Yasar et al. [6]	Y	Y	Y	Y	Y	Y	Y	Y	Y	Y	Include
Lee et al. [18]	Y	Y	Y	Y	Y	Y	Y	Y	Y	Y	Include
Kim et al. [1]	Y	Y	Y	Y	Y	Y	Y	Y	Y	Y	Include
Han et al. [8]	Y	Y	Y	Y	Y	Y	Y	Y	Y	Y	Include

Appendix D 

**Table 7:** Data from 34 articles on partial unilateral lentiginosis characteristics in reported patients

Article authors	No of patients	No of females	No of males	Age	Onset age	Origin	Lesion type	Lesion size	Localization	Other manifestations	Associated conditions
Hughes et al. [26]	1	0	1	23	infant	Black	Light brown macules	5-15 mm	Left C4-T5 dermatomes	Café-au-lait spots	Sickle cell anemia
Trattner et al. [5]	9	5	4	5-16	1-15	Israel	Macules	Small	Neck, face, arms, chest	CALM (2), vitiligo, cutis marmorate	Celiac, asthma
Holder et al. [10]	1	1	0	43	Congenital	UK	Macules, nodules	Small	Right leg	Blue naevi within PUL	Nerve palsy, RBBB
Goldberg [22]	Reply	-	-	-	-	-	-	-	-	-	-
Parslew et al. [13]	1	1	0	14	5	UK	Macules	Small	Left neck/shoulder; right face	None	None
Piqué et al. [3]	7	3	4	13-52	Childhood	Spain	Macules, papules	1-15 mm	Hemisomatic (mostly left)	CALM (3), vitiligo, axillary freckles	Mononeuropathy, anemia
Lee et al. [21]	1	1	0	20	15	Korean	Macules	Rice sized	Right face, trunk, arm	CALM, Lisch nodules	Segmental NF1
Langenbach et al. [29]	1	0	1	5	Birth	Germany	Macules, plaques	0.3-4 cm	Left face, neck, chest, arm	CALM, melanocytic naevi	Astigmatism, myopia
Schaffer et al. [9]	1	1	0	30	5	Peruvian	Macules	1.5-2.5 mm	Left face (V1, V2)	Conjunctival pigmentation	Myopia
Romiti et al. [2]	2	0	2	7, 20	Birth	Brazil, Ger	Maculae	Small	Left neck to leg; right neck to trunk	None	None
Chen et al. [16]	1	1	0	9	Infancy	Taiwanese	Macules	1-2 mm	Left face	CALM, Lisch, axillary freckling	Segmental NF1
Kim et al. [14]	1	0	1	60	Birth	Korean	Macules	Extensive	Left face, neck, trunk, arm	None	None
Palleschi et al. [4]	1	0	1	39	Infancy	Italy	Macules	Small	Left neck, chest, back, arm	Midline crossing	None
Serarslan [25]	1	0	1	22	2	Turkish	Macules	0.1-0.5 cm	Right face	Iris and caruncula nevus	None
Happle [11]	Comment	-	-	-	-	-	-	-	-	-	-
Yu et al. [27]	1	1	0	5	3 mo	Japanese	Macules, papules	1-3 mm	Left arm, shoulder, back	Nevus achromicus	None
In and Kang [15]	1	0	1	39	Childhood	Korean	Macules	2-3 mm	Lower left trunk	Colocalized nevus depigmentosus	None
González-sixto [28]	1	1	0	25	Birth	Spanish	Macules	Small	Right face (V1-V2)	Ocular scleral pigmentation	Nevus of Ota
Lee et al. [18]	10	9	1	23-46	3-26	Korean	Macules	Facial macules	Face mostly cheeks	None	None
Pretel et al. [23]	2	2	0	13, 14	Birth, 5	Spain, Ecu	Macules	1-5 mm	Case 1: L dace; Case 2: R face	None	None
Yasar et al. [6]	16	13	3	15-48	Birth/acq	Turkish	Macules	1-5 mm	Trunk (56%), face/neck	CALM, Lisch, Freckling	Sarcoidosis, breast CA
Kim et al. [19]	1	1	0	19	Early child	Korean	Macules	1.5-2.5 mm	Left cheek, periorbital	None	None
Gupta et al. [7]	1	1	0	21	16	Indian	Macules	0.1-0.5 cm	Right face (V1, V2)	Bulbar conjunctival macules	Seizures
Erdem et al. [20]	1	1	0	28	27	Turkish	Macules	2-3 mm	Right face, scapula, arm, leg	Lisch nodules	Pilocystic astrocytoma
Kim et al. [1]	32	26	6	Mean 27	0-31	Korean	Macules	facial/neck	Face, neck	Interlesional hyperpigment	SLE, RA, cleft lip
Khalil et al. [12]	1	1	0	13	Childhood	USA	Macules	-	Right scapula, cheek	Axillary freckling	None
Hindritiani et al. [17]	1	1	0	35	15	Indon	Macules	-	Forehead, right cheek	Spectrophotometry shift	None
Lueangarun et al. [24]	1	1	0	30	Early child	Thai	Macules	1.5-2.5 mm	Perioirbital, right cheek	None	None
Han et al. [8]	58	39	19	Median 6.6	6.6	Korean	Macules	Agminated	Head/neck, trunk	None	None
González-Sixto et al. [30]	1	1	0	86	Childhood	Spain	Multiple small lentigines; non-pigmented plaque; hyperpigmented macule	Plaque: 2 x 1.8 cm; macule: 5 x 5 mm	Left preauricular area, left face, right arm, right trunk, left leg	Superficially spreading melanoma; melanoma in situ	Routine laboratory and imaging studies normal; No NF stigmata
Lee et al. [31]	1	1	0	28	15 years	Korea	Numerous light-brown macules; café-au-lait macules (CALMs)	Macules: 1-5 mm in diameter	Right cheek, right upper thorax, axillae, back (bilateral)	21 café-au-lait macules (right thorax, arms, back, sacrum)	Otherwise in good health; No NF stigmata; medical/family history negative
Lee et al. [32]	1	1	0	8	Birth (pigment); age 4 (alopecia)	South Korea	Dark-brownish macules; light-brownish patches (CALMs)	Discrete, small macules (size not specified)	Scalp, neck, trunk, and back (bilateral peppered distribution)	4 café-au-lait macules; alopecia universalis	Normal mental/behavioral performance; no NF stigmata; family history negative
Dilek Bayramgürler et al. [33]	1	0	1	33	Birth (pale mark); macules appeared in last 10 years	Turkey	Hypopigmented patch; multiple hyperpigmented macules	Macules: 1-2 mm in diameter	Right neck, back, proximal arm, and right scapula (anterior/posterior)	Nevoid hypopigmentation (nevus depigmentosus)	No extracutaneous or neurological abnormalities
Kabashima et al. [34]	1	1	0	23	Infancy	Japan	Multiple hyperpigmented macules, café au lait spots, hypopigmented background	Macules: 1-2 mm; CALMs: >15 mm	Right upper arm, right upper trunk (shoulder to scapula)	Incomplete hypopigmented area, 4 café au lait spots	Family history positive for similar disorder; no NF stigmata

Appendix E 

**Table 8:** Treated partial unilateral lentiginosis cases reported throughout the literature from 2001 until 2026

Study	Year	n	Age (years)	sex	Origin	Clinical presentation	Treatment	Parameters	Sessions	Outcome
[9]	2001	1	30	F	Peruvian	Brown macules, L upper face	Topicals, cryotherapy, multiple lasers	Not specified	Not specified	Treatment failure, recurrence, transient hypopigmentation
[25]	2007	1	22	M	Turkish	Right facial involvement	cryotherapy	5 s freeze/lesion	4	Successful removal
[18]	2012	10	23-46	9 F/1 M	Korean	Facial PUL	QS Nd:YAG 1064 nm	1.5–2.5 J/cm², 7 mm, every 2 weeks	5-9	>50% improvement, slight recurrence
[23]	2013	2	13-14	F	Not specified	Face, neck, thorax	QS Alexandrite	5–6 J/cm²	3	Substantial to moderate improvement, transient PIH
[19]	2017	1	19	F	Korean	Left cheek, periorbital	QS Nd:YAG 1064 nm	2.5–3 J/cm²	10	Successful, no side effects
[1]	2021	16	0-31	F>M (4:3:1)	Korean	Multiple unilateral lentigines	Mixed lasers	Variable	Avg 4.8	>50% improvement in majority
[17]	2023	1	35	F	Indonesian	Dermatomal facial macules	CuBr laser	511/578 nm	2	Complete clearance, no SE
[24]	2024	1	30	F	Thai	Right cheek, periorbital	532-nm Nd:YAG FPL	0.2–0.4 J/cm²	4	Full improvement, no SE
[8]	2024	Not specified	Not specified	Not specified	Korean	Not specified	Mixed modalities	Not specified	Avg 3	Poor response
[30]	2008	1	86	Female	Spain	Multiple small lentigines since childhood (face, arm, trunk, leg); non-pigmented plaque on left preauricular area; 5-mm hyperpigmented macule on left leg.	Surgical excision	5-mm surgical margin	1 per lesion	No recurrence or new malignancy after 12 months follow-up
[32]	2012	1	8	Female	South Korea	Alopecia universalis; numerous dark-brownish macules since birth on scalp, neck, and trunk; 4 café-au-lait macules.	Topical minoxidil and tacrolimus	3% minoxidil; 0.1% tacrolimus ointment	Just initiated	Results not yet reported
